# The AGE–RAGE Pathway in Endometriosis: A Focused Mechanistic Review and Structured Evidence Map

**DOI:** 10.3390/ijms27031396

**Published:** 2026-01-30

**Authors:** Canio Martinelli, Alfredo Ercoli, Francesco De Seta, Marcella Barbarino, Antonio Giordano, Salvatore Cortellino

**Affiliations:** 1 Sbarro Institute for Cancer Research and Molecular Medicine and Center of Biotechnology, College of Science and Technology, Temple University, 1900 N 12th St, Philadelphia, PA 19122, USA; canio.martinelli@shro.org (C.M.);; 2 Unit of Obstetrics and Gynecology, Department of Human Pathology of Adult and Childhood “Gaetano Barresi”, University of Messina, Via Consolare Valeria 1, 98124 Messina, Italy; 3 Department of Obstetrics and Gynaecology, IRCCS San Raffaele Scientific Institute, University Vita and Salute, 20132 Milan, Italy; 4 Department of Medical Biotechnology, University of Siena, Via Aldo Moro 2, 53100 Siena, Italy; marcella.barbarino@unisi.it; 5 Clinical and Translational Oncology, Scuola Superiore Meridionale (SSM), 80138 Naples, Italy; 6 SHRO Italia Foundation ETS, 10060 Turin, Italy

**Keywords:** AGEs, inflammation, endometriosis, nutrition, RAGE

## Abstract

High Mobility Group Box 1 (HMGB1) and S100 proteins are major ligands of Receptor for Advanced Glycation End-products (RAGE) and have causal roles in endometriosis lesions. Yet the AGE–RAGE pathway that unifies Advanced Glycation End-products (AGEs) with these ligands has not been assessed in endometriosis. In diabetes, atherosclerosis, and chronic kidney disease, AGE–RAGE links insulin resistance and oxidative stress to inflammation, fibrosis, and organ harm. Endometriosis shares key drivers of AGE accumulation, including insulin resistance, oxidative stress, and chronic inflammation. Endometriosis is also linked to higher vascular risk and arterial stiffness. We asked whether AGE–RAGE could bridge metabolic stress to pelvic lesions and systemic risk. We did a focused review of mechanisms and an evidence map of studies on AGEs, RAGE, or known RAGE ligands in endometriosis. We grouped findings as most consistent with a driver, amplifier, consequence, or parallel role. We included 29 studies across human samples, cell systems, and animal models. Few studies measured AGE adducts directly. Most work tracked RAGE ligands (mainly HMGB1 and S100 proteins) and downstream immune and angiogenic programs. Across models, this pattern fits best with a self-reinforcing loop after lesions form. RAGE expression often aligned with lesion remodeling, especially fibrosis. Blood and skin readouts of AGE burden were mixed and varied by cohort and sample type. A central gap is receptor proof. Many models point to shared Toll-like receptor 4 (TLR4)/ nuclear factor kappa B (NF-κB) signaling, but few test RAGE dependence. Overall, current evidence supports AGE–RAGE as a disease-amplifying loop involved in chronic inflammation and fibrosis rather than an initiating trigger. Its effects likely vary by stage and site. Priorities now include direct lesion AGE measurement, paired systemic–pelvic sampling over time, receptor-level studies, and trials testing diet or drug interventions against clear endpoints. Outcomes could include fibrosis, angiogenesis, immune state, pain, and oocyte and follicle function.

## 1. Introduction

Current endometriosis management fails to meet patient needs [1]. Care-seeking patterns document this failure: patients “doctor shop” across multiple specialists, 83.8% experiment with dietary modifications, and 58.8% use supplements which have been identified predominantly through social media rather than clinical guidance [2,3]. While patients accept substantial financial burdens for unvalidated interventions, companies aggressively commercialize the therapeutic vacuum [4,5].

This phenomenon reflects a fundamental reality: endometriosis remains not fully characterized biologically despite affecting 190 million women globally [1]. We cannot predict disease progression. We cannot explain why anatomically similar lesions produce disparate symptoms. We cannot identify therapeutic responders. These represent the basic aspects of natural history that guide management in other chronic diseases [1]. Whereas in oncology and rheumatology, molecular pathway elucidation supports biomarker-driven therapeutic decision-making [6,7], endometriosis management remains predominantly empirical, with clinically significant treatment responses observed in only about fifty percent of patients [1]. Therefore, diagnostic delays average 6.8 years [8].

The consequences extend beyond the pelvis. While physicians struggle with diagnosis and treatment, patients develop systemic complications that defy gynecological explanation. Danish registry data from 60,508 women with endometriosis revealed unexpected cardiovascular vulnerability. Heart attacks and strokes increased 15%. Arrhythmias rose 21%. Heart failure climbed 11% [9]. Most revealingly, multiple studies suggest that, in at least a subset of women, endometriosis is accompanied by a metabolic stress phenotype, including insulin resistance, oxidative stress, and chronic low-grade inflammation, although the strength and consistency of this signal vary across cohorts and covariate adjustments [10,11,12]. Importantly, even in the absence of overt systemic metabolic dysfunction, the lesional and peritoneal microenvironment exhibits oxidative and inflammatory stressors that provide sufficient biochemical conditions for AGE accumulation and RAGE ligand-dependent signaling.

This convergence provides a critical direction. In diabetes and atherosclerosis, the same metabolic disfunction pattern drives end-organ damage through specific molecular mediators that have been successfully characterized and targeted, transforming empirical management into mechanism-based medicine [13,14]. The shared metabolic phenotype suggests that the same molecular pathways may yield therapeutic targets in endometriosis.

Among mediators linking metabolic dysfunction to tissue damage, AGEs represent primary candidates for investigation. AGEs accumulate precisely under the metabolic conditions observed in endometriosis, insulin resistance, oxidative stress, and chronic inflammation, and mediate the same complications now documented in affected women [14]. Preliminary evidence supports this mechanistic parallel. Established RAGE ligands, including S100 proteins and HMGB1, already demonstrate causal involvement in endometriotic pathophysiology [15,16]. Initial studies report elevated systemic AGE markers that correlate with disease severity [17,18].

Yet the nature of this involvement remains undefined. Does AGE–RAGE signaling drive endometriosis pathogenesis? Does it accumulate as a consequence of chronic disease? Might it function as an accessory mechanism (not causative) by amplifying inflammation and tissue damage once disease establishes? Or does it represent a parallel metabolic insult, present for independent reasons yet detrimental to disease course?

This narrative review examines available evidence to address these questions. We first establish the biochemical and pathophysiological foundations of AGE–RAGE biology, then systematically evaluate studies linking this axis to endometriosis. Our aim is to determine which of these mechanistic relationships current data support and to identify the critical gaps requiring investigation.

## 2. Materials and Methods

**Figure 1:**
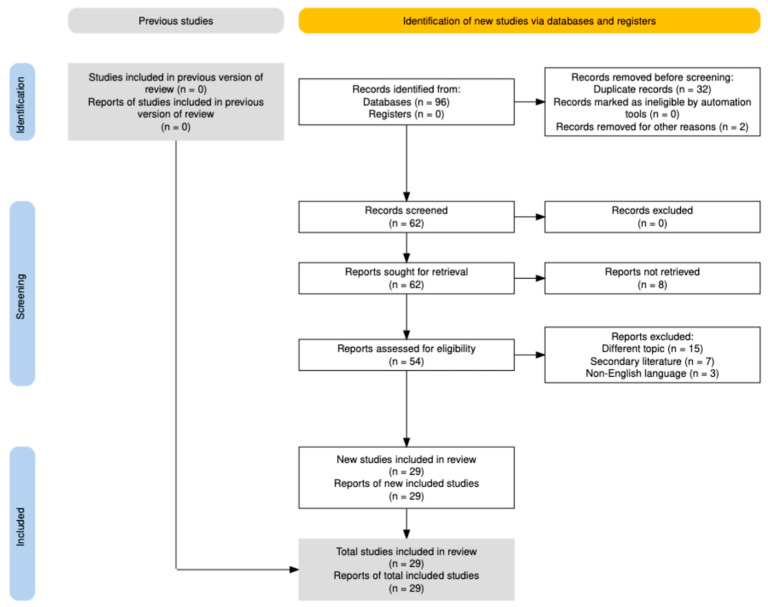
PRISMA flowchart showing study selection process.

This narrative review comprises two methodologically distinct components. The molecular biology of the AGE–RAGE pathway was constructed from the seminal literature selected through author expertise, emphasizing authoritative reviews and landmark mechanistic studies on diabetes, cardiovascular disease, and chronic kidney disease research. Evidence for AGE–RAGE involvement in endometriosis was identified through systematic database searching and is reported in accordance with PRISMA 2020 guidelines (Figure 1), following the systematic–narrative hybrid approach described by Turnbull et al. [19]. This methodology integrates systematic search procedures with narrative synthesis, ensuring comprehensive and transparent literature coverage, while maintaining the interpretive flexibility appropriate for mechanistic and exploratory reviews. The systematic–narrative hybrid approach is well-suited to evidence mapping in emerging fields, where study designs are heterogeneous and meta-analysis is not feasible. The PRISMA 2020 checklist is provided in Supplementary Document S1 for transparency, with items not applicable to narrative synthesis clearly indicated.

### 2.1. Search Strategy

PubMed/MEDLINE and Scopus were searched from January 2000 to October 2025. For PubMed:

((“Endometriosis” [MeSH] OR “Endometriosis” [Title/Abstract] OR “Endometriotic” [Title/Abstract] OR “Endometrioma” [Title/Abstract]) AND (“Advanced glycation end product” [MeSH] OR “Maillard Reaction” [MeSH] OR “Receptor for Advanced Glycation End Products” [MeSH] OR “Advanced glycation end product*” [Title/Abstract] OR “RAGE” [Title/Abstract] OR “Glycation” [Title/Abstract] OR “Carboxymethyl lysine” [Title/Abstract] OR “CML” [Title/Abstract] OR “Methylglyoxal” [Title/Abstract] OR “Pentosidine” [Title/Abstract] OR “S100A8” [Title/Abstract] OR “S100A9” [Title/Abstract] OR “HMGB1” [Title/Abstract])).

For Scopus:

(TITLE-ABS-KEY (endometriosis) AND TITLE-ABS-KEY (advanced glycation end product) OR TITLE-ABS-KEY (advanced glycation end product receptor) OR TITLE-ABS-KEY (maillard reaction) OR TITLE-ABS-KEY (pentosidine) OR TITLE-ABS-KEY (Carboxymethyl lysine) OR TITLE-ABS-KEY (CML) OR TITLE-ABS-KEY (Methylglyoxal) OR TITLE-ABS-KEY (S100A8) OR TITLE-ABS-KEY (S100A9) OR TITLE-ABS-KEY (HMGB1)).

### 2.2. Eligibility Criteria

We included original research investigating AGEs, RAGE, or established RAGE ligands (S100 proteins, HMGB1) in endometriosis. We excluded secondary literature (reviews, meta-analyses, and commentaries), conference abstracts, editorials without original data, correction notices, and studies lacking clear connection to AGE–RAGE biology.

### 2.3. Screening and Selection

The initial search yielded 96 records (51 from Scopus, 45 from PubMed). Two correction articles were removed (one from each database). Records were exported to Rayyan (QCRI) for de-duplication, which identified 32 overlapping articles, yielding 62 unique records for screening.

Following title and abstract screening, 62 reports were sought for full-text assessment. Eight reports could not be retrieved. Of the 54 reports assessed, 25 were excluded for the following reasons: different topic with no relevant connection to AGE–RAGE biology (*n* = 15), secondary literature including reviews and meta-analyses (*n* = 7), and non-English language text (*n* = 3). Twenty-nine original studies were included in the final synthesis.

### 2.4. Data Synthesis

Studies were categorized thematically: direct AGE measurements, RAGE and soluble RAGE expression, RAGE ligand investigations, mechanistic and intervention studies, and clinical correlations. Given evidence heterogeneity and absence of randomized controlled trials, narrative synthesis was employed. Formal quality scoring was not performed, given the exploratory nature of this field.

## 3. Results

### 3.1. Molecular Biology of the AGE–RAGE Pathway

**Figure 2:**
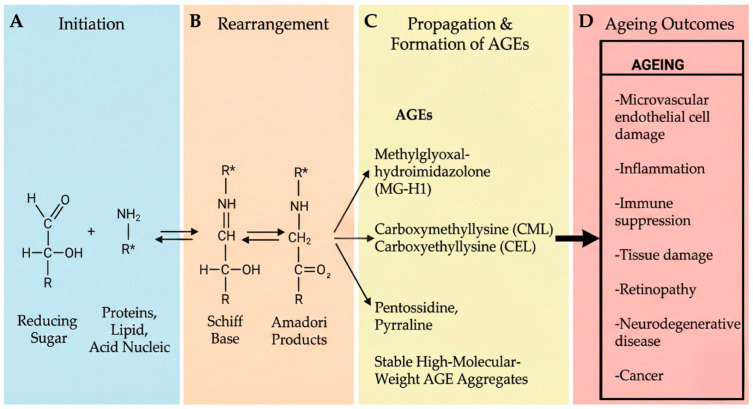
The synthesis of AGEs via the Maillard reaction. Carbonyl groups in reducing sugars interact with free amino groups in amino acids, particularly lysine and arginine, as well as lipids and nucleic acids. This interaction forms a Schiff base, which rearranges into stable ketoamines called Amadori products, which undergo further reactions, notably oxidation, producing dicarbonyl compounds. The dicarbonyls participate in dehydration, condensation, oxidation, or fragmentation reactions, forming AGEs such as methylglyoxal-hydroimidazolone (MG-H1), carboxymethyllysine (CML), carboxyethyllysine (CEL), pentosidine, and pyrraline, and stable high-molecular-weight AGE aggregates.

**Formation and Accumulation of AGEs.** AGEs form through non-enzymatic reactions between reducing sugars and the amine groups of proteins, nucleic acids, and lipids [20]. This process, termed the Maillard reaction, proceeds through defined chemical stages [21]. In the initial phase, carbonyl groups from reducing sugars such as glucose and fructose react with free amino groups, particularly those of lysine and arginine residues, to form a Schiff base [22]. This unstable intermediate rearranges to produce more stable ketoamines known as Amadori products. Amadori compounds then undergo oxidation, generating reactive dicarbonyl intermediates including methylglyoxal, glyoxal, and 3-deoxyglucosone [23,24]. These dicarbonyls participate in further reactions (dehydration, condensation, oxidation, or fragmentation) to yield stable AGE species such as Nε-carboxymethyllysine (CML), Nε-carboxyethyllysine (CEL), pentosidine, pyrraline, and methylglyoxal-hydroimidazolone (MG-H1) [25]. The irreversibility of AGE formation carries important biological consequences. AGEs preferentially accumulate on long-lived proteins including fibrinogen, albumin, and structural matrix components [26]. Collagen and elastin are particularly susceptible due to their longevity and high lysine and arginine content [27,28]. Extracellular matrix proteins undergo AGE modification with accumulation in blood vessel walls and organ parenchyma [29]. CML, one of the most extensively studied AGE species, has been detected in diverse tissues and contributes to the pathogenesis of diabetic complications, atherosclerosis, renal failure, pulmonary fibrosis, and neurodegenerative disease [30,31,32,33,34,35,36,37]. Pentosidine, a fluorescent cross-linking AGE, serves as a marker of cumulative glycoxidative stress and correlates with vascular wall thickening in diabetic patients [38] (Figure 2).

AGEs also possess catalytic properties that extend their biological impact. AGE-modified proteins bind transition metal ions such as Cu^2+^ and Fe^2+^, creating sites that generate reactive oxygen species (ROS) and reactive nitrogen intermediates [39]. Additionally, AGE formation frequently occurs alongside lipid oxidation, producing advanced lipoxidation end products that share receptor-binding properties with classical AGEs [37,40]. These catalytic and oxidative activities amplify the pathogenic potential of AGE accumulation beyond direct protein modification.

**RAGE Receptor Structure and Ligand Diversity.** RAGE transduces signals from AGEs and other ligands into cellular responses. RAGE is a transmembrane protein of the immunoglobulin superfamily encoded by the *AGER* gene [41]. Under physiological conditions, RAGE expression remains low in most adult tissues, with constitutive high expression limited primarily to the lung [42]. However, RAGE is inducible: expression increases substantially in contexts of chronic inflammation, cellular stress, and disease [43,44]. Monocytes, macrophages, endothelial cells, dendritic cells, and various parenchymal cell types express RAGE upon appropriate stimulation [42,45].

A defining feature of RAGE is its capacity to bind structurally diverse ligands. Beyond AGE compounds such as CML and hydroimidazolones [46], RAGE functions as a pattern recognition receptor for damage-associated molecular patterns (DAMPs) and other endogenous molecules. These ligands include HMGB1, members of the S100/calgranulin family of calcium-binding proteins and amyloid-β fibrils [47,48,49]. This broad ligand repertoire positions RAGE as an integrative sensor of tissue damage and metabolic stress rather than a receptor specific to glycation products.

Soluble RAGE (sRAGE) isoforms add regulatory complexity to this system. sRAGE arises through proteolytic cleavage of the membrane-bound receptor by metalloproteinases. A second soluble form, endogenous secretory RAGE (esRAGE), results from alternative splicing of the *AGER* gene [50]. Both isoforms retain extracellular ligand-binding domains but lack transmembrane and intracellular signaling components. As a result, sRAGE and esRAGE function as decoy receptors: they sequester circulating ligands, prevent engagement with membrane-bound RAGE, and attenuate downstream signaling [51,52]. The ratio of membrane-bound to soluble RAGE therefore modulates net pathway activity.

**Figure 3:**
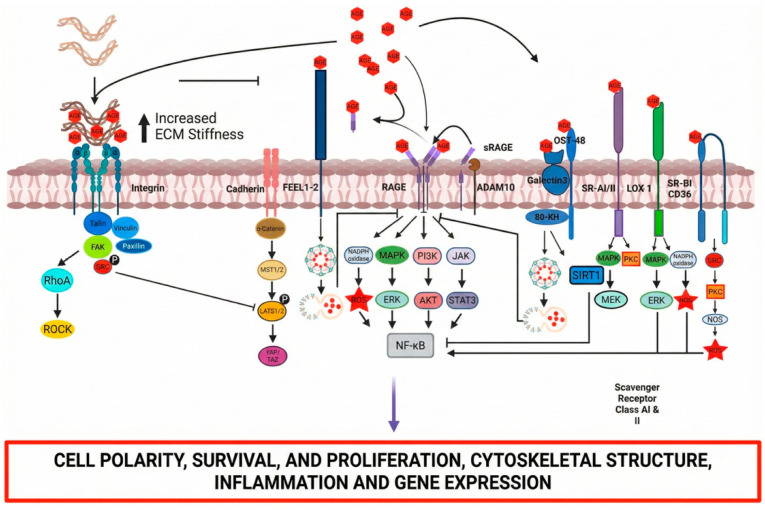
The signaling pathways and mechanisms regulated by AGEs. AGEs influence cellular function by modulating integrins or AGE receptors, including RAGE, OST-48, 80 K-H, galectin-3, SR-A, SR-BI, and LOX-1. AGEs disrupt integrin function by increasing matrix rigidity and altering ECM composition, promoting YAP-TAZ signaling pathway activation. AGE interaction with RAGE activates signaling pathways, including NF-κB, TGF-β, Jak-STAT, and PI3K-Akt, which facilitate inflammation, apoptosis, autophagy, angiogenesis, and ROS production via NADPH oxidase. The AGE receptor components (OST-48/AGE-R1, 80 K-H/AGE-R2, and galectin-3/AGE-R3) facilitates AGE degradation through endocytosis and reduces inflammation by activating SIRT1. The class A scavenger receptor (SR-A), upon activation by AGEs, initiates intracellular signaling pathways, including protein kinase C (PKC) or MAP kinase (MEK), resulting in gene transcription and the release of inflammatory cytokines. AGEs also activate the LOX-1 receptor (lectin-like oxidized low-density lipoprotein receptor 1), inducing inflammation via the MAPK-ERK-regulated pathway and oxidative stress via NADPH oxidase activation. AGEs activate SR-BI and CD36 receptor, leading to oxidative stress through SRC-PKC-NOS pathway. *FEEL-1* (fasciclin, EGF-like, laminin-type EGF-like, and link domain-containing scavenger receptor-1) is implicated in AGE degradation via endocytosis.

**Intracellular Signaling Cascades.** Ligand binding to membrane-bound RAGE initiates intracellular signaling through multiple parallel pathways (Figure 3).

Receptor activation engages mitogen-activated protein kinases (ERK1/2, p38, and SAPK/JNK), STAT3, phosphatidylinositol 3-kinase/Akt, and Rho family GTPases (Rac1, Cdc42) [51,53,54]. These cascades converge on NF-κB, which translocates to the nucleus upon activation [51,53,54].

NF-κB-dependent transcription produces effector molecules central to tissue inflammation and remodeling. Target genes include pro-inflammatory cytokines, the adhesion molecules ICAM-1 and E-selectin, the vascular endothelial growth factor (VEGF), and matrix metalloproteinases [51,53,54]. NF-κB also drives the transcription of RAGE itself, establishing a positive feedback loop: receptor activation amplifies receptor expression, which sustains and intensifies signaling [51,53,54].

RAGE signaling intersects with innate immune activation through the NLRP3 inflammasome. NF-κB activity primes NLRP3 expression, and RAGE-dependent signaling promotes inflammasome assembly [55]. Activated NLRP3 triggers caspase-1 cleavage, which processes pro-IL-1β and pro-IL-18 into their mature secreted forms [56]. This pathway directly links AGE–RAGE activation to the release of potent pro-inflammatory cytokines.

A central consequence of AGE–RAGE-NF-κB signaling is ROS generation [57]. Elevated ROS levels deplete cellular antioxidant defenses, creating a state of intracellular redox imbalance and mitochondrial dysfunction [58,59]. Critically, ROS also activate stress-responsive kinases including MAPK, ERK, IKK, p38, and JNK [60]. Because these kinases feed back into NF-κB activation and RAGE expression, a self-reinforcing cycle emerges: inflammation generates oxidative stress, which sustains inflammation [60]. This positive feedback architecture explains why AGE–RAGE signaling, once initiated, can persist and amplify even after the original stimulus diminishes.

**Direct Effects on Extracellular Matrix.** Distinct from receptor-mediated signaling, AGEs exert direct structural effects on tissues through protein cross-linking. Collagen and elastin, the principal structural proteins of the extracellular matrix, are long-lived molecules with abundant lysine and arginine residues that render them susceptible to glycation [27,28]. AGE-induced cross-links alter the tertiary protein structure, reducing elasticity and increasing tissue stiffness [61,62]. In collagen-rich tissues such as ligaments, tendons, and bones, this manifests as structural weakening and impaired mechanical function [63].

AGE modification also disrupts the matrix turnover. Glycated collagen resists degradation by matrix metalloproteinases, impairing the physiological remodeling required to maintain tissue homeostasis [64]. The persistence of AGE-modified matrix proteins creates a reservoir of RAGE ligands within the extracellular space. This reservoir provides sustained receptor activation even in the absence of ongoing AGE formation and contributes to “metabolic memory”: the phenomenon wherein prior glycoxidative damage continues to drive pathology after the original metabolic insult has resolved [65].

**Biological Outputs of AGE–RAGE Activation.** The molecular events downstream of AGE–RAGE activation converge on several biological processes implicated in chronic disease.

Inflammation represents a primary output. NF-κB-dependent transcription induces pro-inflammatory cytokines including IL-1β, IL-6, and TNF-α [66,67]. Expression of adhesion molecules facilitates leukocyte recruitment to sites of AGE accumulation: ICAM-1 through NF-κB transcriptional activation [51,53,54] and VCAM-1 by promoting monocyte migration [68]. The NLRP3 inflammasome pathway amplifies cytokine release and sustains inflammatory signaling [55,56,69].

Oxidative stress constitutes both a downstream effect and a feed-forward amplifier. ROS generation creates a pro-oxidant cellular environment with mitochondrial dysfunction [57,58,59]. Oxidative modification of proteins and lipids generates additional RAGE ligands, perpetuating the activation cycle [37,39,40].

Fibrosis emerges from coordinated matrix production and impaired degradation. AGE–RAGE signaling activates TGF-β pathways and promotes the transcription of extracellular matrix genes, including those encoding collagen and laminin [70,71,72]. Combined with AGE cross-link-mediated resistance to matrix degradation, these changes favor net matrix accumulation and progressive tissue fibrosis.

Angiogenesis results from VEGF transcription downstream of NF-κB [51,53,54]. In pathological contexts, this promotes endothelial proliferation, new vessel formation, and lesion vascularization.

Cellular dysfunction manifests variably depending on the cell type and signaling intensity. Sustained RAGE activation can induce cellular senescence or apoptosis [43,44]. Mitochondrial damage from oxidative stress impairs cellular energetics and compromises viability [58,59].

**HMGB1 and S100 Proteins as RAGE Ligands.** Although AGEs represent classical RAGE ligands, HMGB1 and S100 proteins engage the same receptor and activate overlapping downstream pathways.

HMGB1 is a nuclear protein that functions intracellularly as a DNA-binding architectural factor. Upon cellular stress, damage, or death, HMGB1 translocates to the cytoplasm and is released into the extracellular space, where it functions as a DAMP [47,48,49]. Extracellular HMGB1 binds RAGE, activating NF-κB and inducing pro-inflammatory cytokine production. HMGB1 also signals through Toll-like receptors, meaning its biological effects are not exclusively RAGE-dependent: A consideration relevant to interpreting studies that measure HMGB1 without directly assessing RAGE.

S100 proteins constitute a family of calcium-binding proteins with diverse intracellular functions. Several members, such as S100A8, S100A9, and S100A12 (also termed EN-RAGE), are released from activated immune cells and function as extracellular pro-inflammatory mediators [47,48,49]. S100A8 and S100A9 frequently form a heterodimer known as calprotectin. Upon release, these proteins bind RAGE and activate NF-κB-dependent inflammatory programs [47,48,49].

The shared receptor usage among AGEs, HMGB1, and S100 proteins has mechanistic implications. Any of these ligands can activate RAGE and drive downstream signaling. Inflammatory conditions that induce HMGB1 or S100 release can therefore amplify the biological effects of AGE accumulation through convergent receptor engagement. This convergence also means that RAGE-dependent pathology may proceed even when AGE levels are modest, provided alternative ligands are present.

**Established Pathophysiological Precedent.** The mechanisms outlined above have been validated across multiple disease contexts, establishing a biological precedent for AGE–RAGE involvement in chronic inflammatory and fibrotic conditions.

In diabetes mellitus, chronic hyperglycemia accelerates AGE formation. AGE–RAGE signaling contributes to nephropathy, retinopathy, neuropathy, and cardiovascular complications [33,65,68,73,74,75,76,77,78,79,80,81,82,83,84,85,86]. Diabetic nephropathy involves AGE accumulation in renal glomeruli, RAGE upregulation in mesangial cells and podocytes, and the activation of inflammatory and fibrotic programs [33,80,84,85,86,87]. Diabetic retinopathy features AGE-induced pericyte apoptosis, blood–retinal barrier disruption, and pathological angiogenesis [88,89,90,91,92,93]. Cardiovascular disease in diabetes is characterized by AGE-mediated vascular stiffening, endothelial dysfunction, and accelerated atherosclerosis [37,38,68,94,95,96,97,98,99,100,101,102,103,104,105,106,107,108,109,110,111,112,113].

Beyond diabetes, the AGE–RAGE pathway has been implicated in age-related vascular disease, chronic kidney disease, pulmonary fibrosis, and neurodegenerative disorders [75,76,77]. The consistent involvement of this pathway across conditions characterized by chronic inflammation, oxidative stress, and tissue remodeling establishes biological plausibility for its role in other diseases sharing these features.

The molecular architecture of the AGE–RAGE pathway, which is characterized by diverse ligand inputs, convergent NF-κB-dependent signaling, and outputs spanning inflammation, oxidative stress, fibrosis, and angiogenesis, provides a mechanistic framework for interpreting findings in endometriosis. The following sections examine evidence for AGE–RAGE pathway activation in endometriosis and evaluate whether this system functions as a disease initiator, amplifier, or parallel marker of tissue pathology.

### 3.2. The AGE–RAGE Pathway in Endometriosis

#### 3.2.1. Characteristics of Included Studies

Our literature search identified 29 original studies published between 2008 and 2025 examining AGE–RAGE pathway components in endometriosis, yielding 451 discrete data extractions for analysis. The publication timeline shows early foundational work (8 studies before 2020), followed by accelerating interest, with 21 studies appearing from 2020 onwards and 9 in 2024–2025 alone (Table 1).

**Figure 4:**
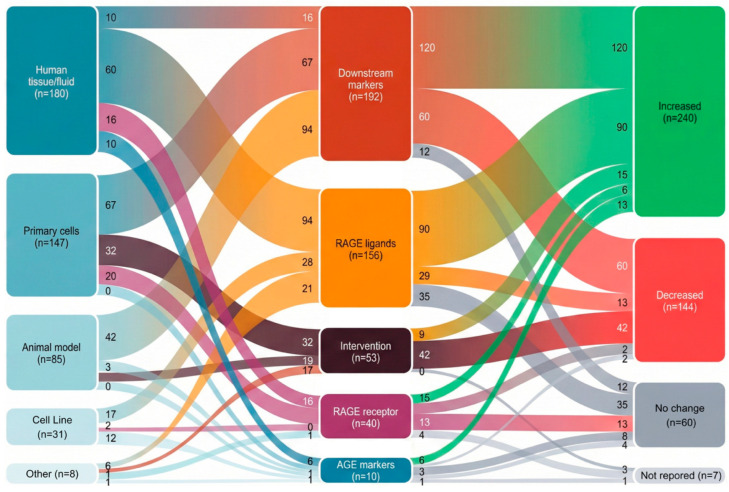
Evidence flow across model systems, axis components, and effect directions in endometriosis AGE–RAGE research. In the alluvial diagram displaying the distribution of 451 data extractions from 29 studies, the left column shows experimental model systems: human tissue or fluid samples (*n* = 180; 39.9%), primary cell cultures (*n* = 147; 32.6%), animal models (*n* = 85; 18.8%), cell lines (*n* = 31; 6.9%), and other approaches (*n* = 8; 1.8%). The middle column shows the AGE–RAGE pathway components examined: downstream markers including inflammatory cytokines and signaling mediators (*n* = 192; 42.6%), RAGE ligands, predominantly HMGB1 and S100 proteins (*n* = 156; 34.6%), intervention studies (*n* = 53; 11.8%), RAGE receptor measurements (*n* = 40; 8.9%), and direct AGE adduct quantification (*n* = 10; 2.2%). The right column shows the effect direction: increased (*n* = 240; 53.2%), decreased (*n* = 144; 31.9%), no change (*n* = 60; 13.3%), or not reported (*n* = 7; 1.6%). Flow width is proportional to the extraction count. Notable patterns: AGE markers exclusively derive from human samples; intervention studies show predominantly decreased effects (79%); and downstream markers show predominantly increased effects (63%).

Study designs were heterogeneous: 10 studies analyzed human clinical samples exclusively, 12 combined human tissue collection with in vitro or in vivo experimental models, and 7 employed experimental systems alone (cell lines or animal models). Among human studies, ovarian endometrioma was the most frequently examined phenotype (18 studies), with deep infiltrating endometriosis (1 study), peritoneal disease, and infertility-associated endometriosis (3 studies) represented in smaller subsets. Five studies did not specify a particular phenotype. Sample sources included human tissue and fluid specimens (180 extractions), primary cell cultures (147 extractions), animal models (85 extractions), and immortalized cell lines (31 extractions) (Figure 4).

The pathway components examined across these studies are distributed as follows: RAGE ligands comprised 156 extractions (34.6%), predominantly HMGB1 (110 extractions), with S100 family proteins including S100A8, S100A9, S100B, and EN-RAGE/S100A12 accounting for 36 extractions. Downstream pathway markers represented the largest category (192 extractions; 42.6%), including inflammatory cytokines (IL-6, IL-1β, TNF-α, and MCP-1), NF-κB signaling components, autophagy markers (Beclin1, ATG13, and LC3-II), and metabolic readouts. Direct RAGE receptor measurements (mRNA, protein, membrane-bound RAGE, or soluble sRAGE) accounted for 40 extractions (8.9%) across 11 studies. AGE adduct markers (carboxymethyllysine, pentosidine, and skin autofluorescence) appeared in only 10 extractions (2.2%) from three studies, representing a notable gap in the evidence base. An additional 53 extractions (11.8%) were derived from intervention experiments in 10 studies testing pharmacological or genetic manipulation of *pathway* components (Supplementary Document S2).

#### 3.2.2. Evidence for AGE Burden in Endometriosis 

Direct measurements of specific AGE adducts in endometriosis compartments remain limited but provide an initial signal consistent with local (pelvic) enrichment rather than strong systemic elevation. Fujii et al. [119] found that CML levels did not differ significantly in plasma or follicular fluid between women with and without endometriosis, yet showed a borderline increase in peritoneal fluid (*p* = 0.05, Mann–Whitney U), a pattern consistent with a localized peritoneal AGE-associated signal.

Subsequent work broadened the systemic burden perspective using non-invasive tissue-based proxies. Women with imaging-confirmed endometriosis exhibited higher skin AGE autofluorescence than matched controls (2.00 ± 0.6 vs. 1.70 ± 0.2; *p* = 0.013), suggesting an elevated cumulative AGE load at the organismal level, although without specifying the underlying AGE species [18].

**Figure 5:**
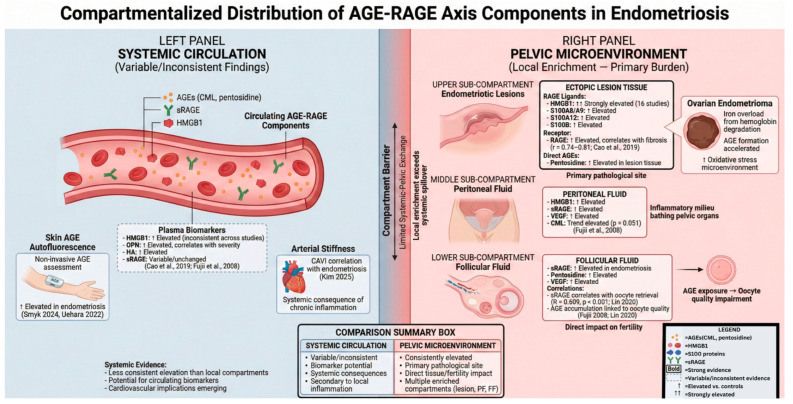
Compartmentalized distribution of AGE–RAGE pathway components in endometriosis: systemic circulation versus pelvic microenvironment [18,117,119,122,124,133]. The AGE–RAGE pathway in endometriosis demonstrates marked compartmentalization, with predominant burden localized to the pelvic microenvironment rather than systemic circulation. ***Systemic circulation (left panel)*** shows variable and inconsistent findings across studies. Plasma biomarkers including HMGB1, osteopontin (OPN), and hyaluronic acid (HA) demonstrate elevation in some but not all studies, with potential utility as circulating biomarkers requiring further validation (Cao et al., 2019) [117]. Plasma sRAGE levels remain largely unchanged. The non-invasive assessment of systemic AGE burden via skin autofluorescence reveals elevated accumulation in women with endometriosis (Smyk et al., 2024; Uehara et al., 2022) [18,133]. Systemic consequences include the correlation with arterial stiffness as measured by the cardio-ankle vascular index (CAVI), suggesting cardiovascular implications of chronic AGE–RAGE activation (Kim et al., 2025) [122]. ***Pelvic microenvironment (right panel)*** demonstrates consistent and pronounced elevation across multiple compartments. Ectopic lesion tissue shows the strongest evidence, with HMGB1 representing the most extensively characterized component (16 studies, 132 data points) alongside S100 family proteins (S100A8/A9, S100A12, and S100B). RAGE receptor expression in lesions correlates strongly with fibrosis severity (r = 0.74–0.81; *p* < 3.2 × 10^−6^) [117]. Peritoneal fluid exhibits elevated HMGB1, sRAGE, and VEGF and trends toward increased carboxymethyl-lysine (CML), reflecting the inflammatory milieu bathing pelvic structures [119]. Follicular fluid demonstrates elevated sRAGE and pentosidine concentrations, with sRAGE levels correlating with the oocyte retrieval number (R = 0.609, *p* < 0.001) [124], implicating direct AGE–RAGE involvement in fertility impairment. Ovarian endometriomas provide a microenvironment of accelerated AGE formation due to iron overload from hemoglobin degradation and heightened oxidative stress. This compartmentalized pattern suggests that, while systemic markers may serve as accessible biomarkers, the primary pathophysiological burden of AGE–RAGE activation is localized to the pelvic cavity, directly impacting lesion biology, peritoneal inflammation, and reproductive function. ***Abbreviations***: AGE, advanced glycation end product; CAVI, cardio-ankle vascular index; CML, Nε-carboxymethyl-lysine; FF, follicular fluid; HA, hyaluronic acid; HMGB1, high-mobility group box 1; OPN, osteopontin; PF, peritoneal fluid; RAGE, receptor for advanced glycation end products; sRAGE, soluble RAGE; and VEGF, vascular endothelial growth factor.

Longitudinal evidence further links AGE biology to cardiometabolic phenotypes in affected women. In a prospective cohort followed across the perimenopausal transition, baseline serum pentosidine correlated with worsening arterial stiffness (ΔCAVI) over time (r = 0.28, *p* = 0.0071) [133]. These data collectively support modest evidence for systemic AGE burden differences (varying by measurement approach) and a possible pelvic-compartment signal but do not establish lesion-specific AGE accumulation as an initiating driver of endometriosis (Figure 5).

#### 3.2.3. RAGE Expression and Localization in Endometriosis 

Multiple studies document RAGE presence across endometriosis-relevant tissues and cells. Fujii et al. [119] detected RAGE transcripts in eutopic endometrium and localized RAGE immunohistochemically in ectopic endometrial cells (including ovarian endometrioma contexts), with additional staining in the vascular endothelium and macrophages.

Ex vivo stromal cell studies support upregulation of the receptor–ligand arm of the pathway in lesion-derived cells. Primary stromal cells from endometriotic lesions demonstrated higher RAGE (*AGER*) and EN-RAGE (*S100A12*) expression at both mRNA and protein levels compared with controls [126]. Related in vitro work indicates that inflammatory stimulation (e.g., LPS) can modulate this pathway and that pharmacologic interventions, including PPAR-γ ligands and statin exposure in LPS-driven settings, can suppress RAGE/EN-RAGE expression alongside broader inflammatory and angiogenic programs in stromal cells [127,128].

#### 3.2.4. RAGE–Fibrosis Association and Lesion Maturation

Human ovarian endometrioma samples revealed a strong positive correlation between lesional fibrosis and RAGE staining (r = 0.74–0.81; *p* < 3.2 × 10^−6^), supporting an association between RAGE expression and fibrotic remodeling in established lesions [117]. Parallel observations in murine transplantation models showed that, as lesions matured, fibrosis increased and again correlated positively with RAGE staining (r = 0.81–0.96; *p* < 1.5 × 10^−4^), consistent with a conserved cross-species relationship once lesions are established.

**Figure 6:**
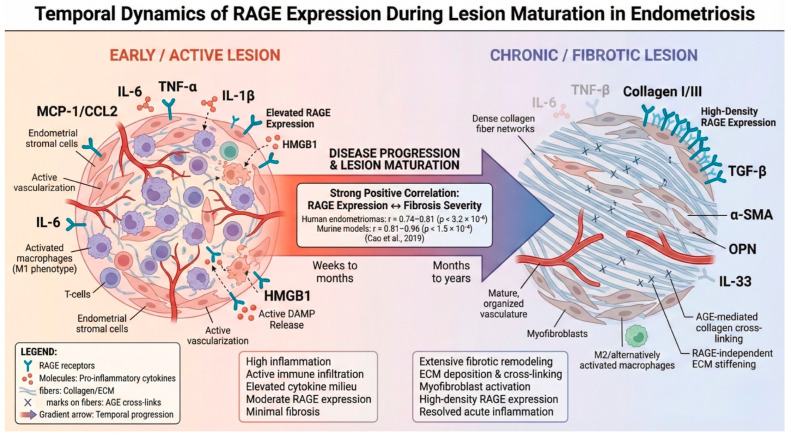
Temporal dynamics of RAGE expression during endometriotic lesion maturation [117]. Endometriotic lesions undergo progressive phenotypic evolution from an early inflammatory state toward chronic fibrotic remodeling. ***Early/active lesions*** (**left** panel) are characterized by abundant immune cell infiltration, elevated pro-inflammatory cytokines (IL-6, TNF-α, IL-1β, and MCP-1), active angiogenesis, and moderate RAGE receptor expression, with ongoing DAMP release (particularly HMGB1) from stressed cells. ***Chronic/fibrotic lesions*** (**right** panel) demonstrate the resolution of acute inflammation, extensive extracellular matrix deposition, collagen accumulation, myofibroblast activation (α-SMA^+^), and markedly increased RAGE receptor density. Fibrotic lesions additionally exhibit the elevated expression of TGF-β, osteopontin (OPN), and IL-33, which cluster positively with RAGE expression, while inflammatory markers (HMGB1, TLR4, and p-p65) are inversely correlated with the extent of fibrosis. ***Abbreviations***: α-SMA, alpha-smooth muscle actin; AGE, advanced glycation end product; DAMP, damage-associated molecular pattern; ECM, extracellular matrix; HMGB1, high-mobility group box 1; IL, interleukin; MCP-1, monocyte chemoattractant protein-1; NF-κB, nuclear factor kappa-light-chain-enhancer of activated B cells; OPN, osteopontin; RAGE, receptor for advanced glycation end products; TGF-β, transforming growth factor beta; TLR4, Toll-like receptor 4; and TNF-α, tumor necrosis factor alpha.

This pattern positions RAGE more strongly as a marker and/or mediator of chronic lesion remodeling, particularly fibrosis, than as a simple early inflammatory “on/off” determinant (Figure 6). 

#### 3.2.5. Immune-Cell RAGE and Disease Severity 

At the immune-cell level, peritoneal macrophage surface RAGE appears to decline with increasing disease severity. Flow cytometry-based phenotyping demonstrated that the macrophage RAGE mean fluorescence intensity negatively correlated with the rASRM score (r = −0.660, *p* < 0.001), alongside similar negative correlations for other pattern-recognition receptors (TLR4, TLR2, and CD36) [130]. Complementary evidence supports RAGE transcriptional responsiveness in peritoneal immune cells. In an ex vivo drug-delivery study, CD14^+^ peritoneal macrophages from women with endometriosis (stages II–IV) demonstrated increased RAGE mRNA expression when exposed to an immunomodulator (GMDP) immobilized on aminopropyl-modified silica nanoparticles compared with medium-only controls (*p* < 0.05); notably, free GMDP and unmodified nanoparticle delivery did not elicit this response, suggesting that nanoparticle surface chemistry can modulate RAGE inducibility in endometriosis-derived peritoneal macrophages [114]. Mechanistically, ex vivo HMGB1 exposure reduced macrophage *RAGE/AGER* expression, including decreased *AGER* mRNA and surface RAGE, supporting a model in which sustained DAMP exposure may contribute to pattern-recognition receptor desensitization or phenotype shifting in advanced disease.

#### 3.2.6. Soluble RAGE in Reproductive and Peritoneal Compartments 

Soluble RAGE findings are compartment-dependent and directionally heterogeneous across cohorts. Fujii et al. [119] found that plasma sRAGE did not differ between women with and without endometriosis, whereas peritoneal fluid and follicular fluid patterns suggested local regulation; peritoneal fluid differences were modest and appeared sensitive to outliers, while follicular fluid sRAGE was higher in the endometriosis group.

Infertility-focused cohorts suggest phenotype-dependent behavior. In an IVF cohort including women with deeply infiltrating endometriosis, follicular fluid sRAGE was reduced and positively correlated with ovarian response: r = 0.609 for the number of oocytes retrieved and r = 0.626 for the number of mature oocytes (both *p* < 0.001), consistent with a potentially protective or decoy-like role of sRAGE in the follicular microenvironment [124].

The soluble RAGE literature thus supports the concept that local ligand-buffering capacity may be clinically relevant in reproductive niches, while emphasizing that sRAGE directionality likely depends on cohort characteristics (e.g., infertility context, lesion phenotype) and analytical sensitivity.

#### 3.2.7. HMGB1 as a Central RAGE Ligand Candidate in Endometriosis 

Among RAGE ligands, HMGB1 is one of the most extensively studied in endometriosis and shows multi-compartment signals across tissue, blood-derived cells, and functional models. Tissue studies report increased HMGB1 in ectopic lesions, particularly ovarian endometrioma, often discussed in relation to inflammatory amplification [117,120,132,135]. Beyond abundance, HMGB1 localization can shift in disease: in ovarian endometrioma cyst walls, staining patterns were consistent with increased extranuclear/cytoplasmic presence and reduced nuclear staining compared with controls, aligning with a DAMP-like pro-inflammatory state [121]. Consistent with these observations, independent work confirmed ectopic lesion-specific HMGB1 enrichment: immunohistochemistry and tissue ELISA demonstrated elevated HMGB1 in ovarian endometrioma compared with both eutopic endometrium from the same patients and control endometrium from women without endometriosis (*p* < 0.001 for each ectopic comparison), while eutopic versus control differences were not significant [120]. HMGB1 levels in ectopic tissue correlated positively with pro-inflammatory cytokines including IL-1β (r = 0.50; *p* = 0.002), IL-6 (r = 0.37; *p* = 0.03), and TNF-α (r = 0.36; *p* = 0.04). Systemic HMGB1 evidence varies depending on whether investigators quantified mRNA (in blood immune cells) versus protein (in serum, peritoneal, or menstrual compartments). HMGB1 mRNA in PBMCs was elevated in women with endometriosis-associated infertility and increased with rASRM stage [115]. By contrast, protein-based comparisons in some cohorts did not show clear case–control discrimination in serum or peritoneal fluid despite marked levels in menstrual blood [129]. These compartment-specific differences highlight why HMGB1 may be biologically important without consistently functioning as a standalone diagnostic biomarker. Across mechanistic models, HMGB1 is repeatedly implicated as an amplifier of lesion-relevant phenotypes. Oxidative stress paradigms increased HMGB1 release and promoted inflammatory signaling in endometrial stromal contexts, with downstream blockade experiments frequently pointing to TLR4 and NF-κB as key mediators [137]. Hypoxic conditions increased HMGB1-related signaling and lesion-relevant cellular behaviors, with HMGB1 perturbation reducing proliferative, invasive, and inflammatory outputs [120]. Metabolic remodeling pathways, including lactate/histone lactylation and post-transcriptional control mechanisms, can enhance HMGB1 expression and support migration, invasion, and lesion growth phenotypes in vitro and in animal transplantation models [118,135]. In the reproductive microenvironment, oxidative stress increased HMGB1 release and inflammatory signaling through TLR4/NF-κB-related pathways in granulosa cell models, again without a direct demonstration of RAGE dependence [122]. HMGB1 has also been linked to nociceptive sensitization pathways in the animal models of endometriosis-associated hyperalgesia, with signaling experiments emphasizing TLR4/MyD88 involvement [131].

**Figure 7:**
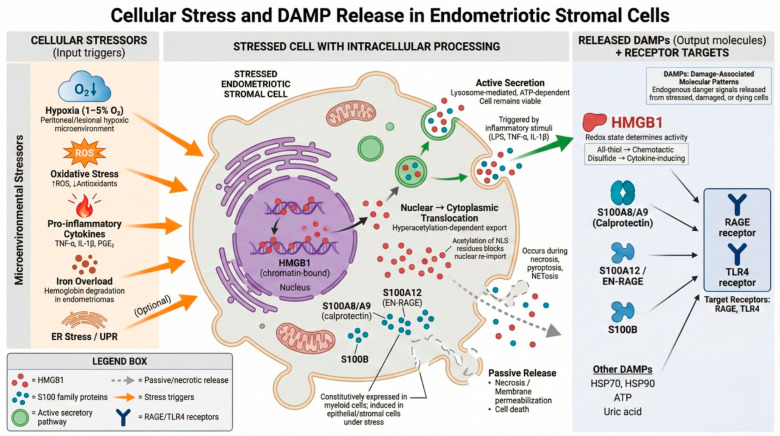
Cellular stress triggers and DAMP release mechanisms in endometriotic stromal cells. Under stress conditions, HMGB1 (high-mobility group box 1), normally a chromatin-associated nuclear protein, undergoes hyperacetylation of lysine residues within its nuclear localization sequences, blocking nuclear re-import and promoting cytoplasmic accumulation. Cytoplasmic HMGB1 is subsequently released into the extracellular space via two distinct mechanisms: (1) active secretion through lysosome-mediated exocytosis, an ATP-dependent process triggered by inflammatory stimuli that preserves cell viability; and (2) passive release during necrotic cell death or membrane permeabilization. The immunological activity of extracellular HMGB1 is determined by its redox state: All-thiol HMGB1 exhibits chemotactic activity, disulfide HMGB1 (C23–C45 disulfide bond) functions as a cytokine-inducing molecule through TLR4 engagement, and fully oxidized (sulfonyl) HMGB1 is immunologically inactive. Additional DAMPs released from stressed endometriotic cells include S100 family members: S100A8/A9 (calprotectin), S100A12 (EN-RAGE), and S100B. These calcium-binding proteins are constitutively expressed in myeloid cells but are induced in epithelial and stromal cells under inflammatory or oxidative stress conditions.Released DAMPs serve as endogenous danger signals that activate pattern recognition receptors, principally RAGE (receptor for advanced glycation end products) and TLR4 (Toll-like receptor 4), initiating downstream inflammatory signaling cascades. HMGB1 constitutes the most extensively characterized DAMP in endometriosis research, contributing 132 data points across 16 studies in the current evidence base. ***Abbreviations***: ATP, adenosine triphosphate; DAMP, damage-associated molecular pattern; ER, endoplasmic reticulum; HMGB1, high-mobility group box 1; IL-1β, interleukin-1 beta; NLS, nuclear localization sequence; PGE_2_, prostaglandin E2; RAGE, receptor for advanced glycation end products; ROS, reactive oxygen species; TLR4, Toll-like receptor 4; TNF-α, tumor necrosis factor alpha; and UPR, unfolded protein response.

HMGB1 knockdown experiments further link HMGB1 to hypoxia-driven inflammatory and autophagy programs. In primary stromal cells from ectopic lesions exposed to hypoxia (1% O_2_), shRNA-mediated HMGB1 silencing reduced secreted HMGB1 and attenuated the hypoxia-induced increase in IL-6, IL-1β, and TNF-α secretion (*p* < 0.01–0.001 versus hypoxia alone); autophagy markers (Beclin1, ATG13, and LC3-II) that were elevated in ectopic tissue and further increased under hypoxia were also reduced by HMGB1 knockdown [120]. Upstream of HMGB1 induction, prostaglandin E2-driven pyroptosis has been implicated as a mechanistic trigger: in an endometrial stromal cell line (hEM15A), PGE2 exposure increased HMGB1 protein expression, an effect attenuated by NLRP3 inhibition (CY-09) or caspase-1 blockade (VX-765); parallel findings in a murine peritoneal implantation model showed elevated HMGB1 immunostaining in lesions from PGE2-treated versus vehicle-treated mice (*p* < 0.05) [11] (Figure 7).

Endometriotic stromal cells within the peritoneal microenvironment are exposed to multiple concurrent stressors that promote damage-associated molecular pattern (DAMP) release. Principal triggers include hypoxia (1–5% O_2_ in lesional tissue), oxidative stress (elevated reactive oxygen species with diminished antioxidant capacity), pro-inflammatory cytokine exposure (TNF-α, IL-1β, and PGE_2_), and iron overload from hemoglobin degradation within endometriomas.

These results position inflammasome/pyroptosis activation as a potential upstream regulator of HMGB1 availability in the endometriosis microenvironment.

#### 3.2.8. Pharmacologic Modulation of the HMGB1-Inflammatory Axis

Pharmacologic intervention studies provide a proof of concept for the therapeutic modifiability of HMGB1-related inflammatory signaling. Sitagliptin, a dipeptidyl peptidase-4 (DPP-4) inhibitor, attenuated hypoxia-induced HMGB1 upregulation in primary human endometrial stromal cells: pretreatment reduced both HMGB1 mRNA and secreted protein in a dose-dependent manner (*p* < 0.0001 for hypoxia effect; *p* < 0.05–0.01 for sitagliptin reversal) [123]. This effect occurred alongside reductions in oxidative stress (decreased ROS, increased GSH), suppression of pro-inflammatory cytokines (TNF-α, IL-6, and MCP-1), and inhibition of both p38 MAPK and NF-κB signaling. While sitagliptin was not tested in an endometriosis animal model, these findings suggest that pharmacologic interventions targeting hypoxia-inflammatory cascades can modulate HMGB1 availability in stromal cell contexts.

Importantly, although HMGB1 is frequently framed as a RAGE ligand, many studies do not directly test RAGE dependence (e.g., via RAGE blockade or knockdown) and instead provide more direct evidence for TLR4/NF-κB as proximate downstream mechanisms. HMGB1 is therefore best interpreted as a biologically active alarmin within the AGE–RAGE ligand repertoire rather than definitive proof of RAGE-mediated signaling in every setting.

#### 3.2.9. S100 Family Ligands and the Broader RAGE Ligand Landscape

S100 proteins constitute a second major ligand class implicated in endometriosis.

EN-RAGE/S100A12 in lesion-derived stromal cells and circulation. Ex vivo stromal cell evidence indicates increased EN-RAGE/S100A12 expression alongside increased RAGE in endometriosis-derived stromal cells [127]. Complementary multiplex inflammation profiling identified higher plasma EN-RAGE in women with endometriosis versus surgical controls (fold change 1.63; *p* = 0.001) [17].

S100A8/A9 in lesions and recurrence risk. Proteomic and transcriptomic profiling of ovarian endometrioma cyst walls identified S100A8 and S100A9 as strongly upregulated, with higher expression associated with postoperative recurrence, supporting relevance to lesion persistence [138].

Pre-diagnostic circulating S100A9 signal. Prospective population-based proteomics provides evidence that circulating S100A9 elevations can precede clinical diagnosis. In a nested case–control analysis, higher pre-diagnostic plasma S100A9 was associated with later laparoscopically confirmed endometriosis (OR 1.52 per SD increase, 95% CI 1.19–1.94), with association strengthening when blood was collected within two years of diagnosis (OR 2.42, 95% CI 1.17–4.98) [125]. Because receptor/pathway usage was not tested, these findings support early systemic innate immune activation involving a RAGE ligand rather than demonstrating RAGE-specific signaling.

S100A9-high macrophages and S100A9 inhibition. Recent mechanistic work attributes a lesion-promoting role to S100A9 in eutopic endometrium: S100A9-high macrophages displayed a ferroptosis-associated transcriptional signature, and in vitro ferroptosis-like stress increased S100A9 expression and secretion through an NF-κB-linked mechanism [136]. Pharmacologic inhibition of S100A9 with tasquinimod reduced endothelial migration and invasion behaviors and decreased lesion burden and angiogenesis proxies (e.g., CD31 staining) in a mouse transplantation model. As emphasized in that study, receptor dependence (RAGE vs. TLR4) was not experimentally resolved.

#### 3.2.10. Downstream Signaling Pathways Repeatedly Linked to AGE–RAGE Biology

**Figure 8:**
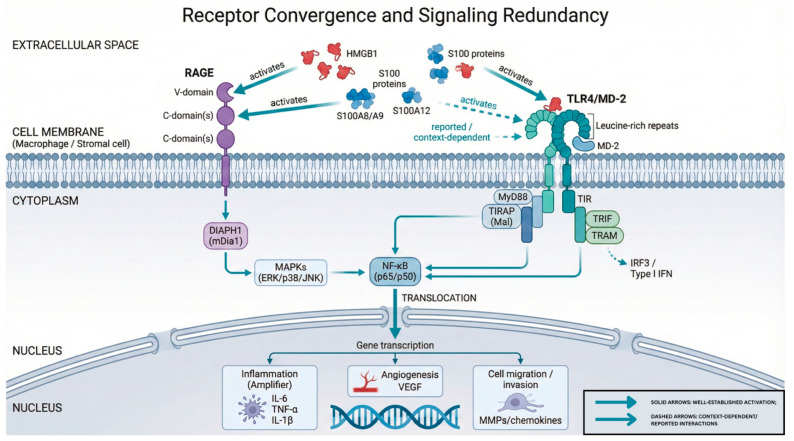
Mechanistic pathway diagram of the AGE–RAGE pathway in endometriosis pathophysiology.AGEs accumulate through endogenous glycation reactions accelerated by hyperglycemia and oxidative stress, while exogenous dietary AGEs (dAGEs) represent an underexplored contributor in endometriosis. Damage-associated molecular patterns (DAMPs), including HMGB1 and S100 protein family members (S100A8/A9, S100A12/EN-RAGE, S100B), serve as additional RAGE ligands released from stressed or necrotic cells. HMGB1 constitutes the most extensively characterized ligand in endometriosis research (132 data points across 16 studies).Full-length RAGE (FL-RAGE) activation triggers the recruitment of adaptor protein DIAPH1 (mDia1), initiating MAPK cascades (ERK1/2, p38, and JNK) that converge on NF-κB (p65/p50) activation. Nuclear translocation of NF-κB drives the transcription of pro-inflammatory cytokines (IL-1β, IL-6, and TNF-α), chemokines (MCP-1/CCL2), adhesion molecules (ICAM-1, VCAM-1), angiogenic factors (VEGF), and, notably, RAGE itself, establishing a positive feedback loop that amplifies inflammatory signaling. This RAGE-NF-κB-RAGE amplification circuit represents the mechanistic basis for the proposed role of AGE–RAGE as a disease amplifier rather than an initiator. sRAGE functions as a protective decoy receptor by sequestering circulating ligands, though its biomarker potential remains underexplored in endometriosis (two studies). TLR4 shares ligand recognition with RAGE for HMGB1 and S100 proteins, with both pathways converging on NF-κB activation; however, the relative contribution and specificity of each receptor in endometriosis remains undefined. HMGB1 additionally modulates autophagy through Beclin1 activation, with context-dependent cytoprotective or pathological outcomes. RAGE-independent mechanisms include direct AGE-mediated protein cross-linking, contributing to extracellular matrix stiffening and fibrosis, though direct evidence in endometriosis tissue is limited.Downstream pathological endpoints encompass inflammation (macrophage activation, peritoneal inflammation), fibrosis (myofibroblast activation, adhesion formation), angiogenesis (VEGF-driven lesion vascularization), and clinical manifestations, including pain (nociceptor sensitization, spinal neuroinflammation), infertility (follicular fluid AGE accumulation, oocyte quality impairment), and emerging systemic cardiovascular effects (arterial stiffness). Red symbols indicate potential therapeutic intervention points: dietary AGE reduction, sRAGE enhancement or RAGE antagonism, and NF-κB pathway inhibition.Solid arrows indicate well-documented pathways; dashed arrows indicate context-dependent or limited evidence; and dashed boxes highlight critical evidence gaps requiring further investigation. ***Abbreviations***: AGE, advanced glycation end product; CML, Nε-carboxymethyl-lysine; DAMP, damage-associated molecular pattern; DIAPH1, diaphanous-related formin 1; ECM, extracellular matrix; ERK, extracellular signal-regulated kinase; FL-RAGE, full-length RAGE; HMGB1, high-mobility group box 1; IFN, interferon; IRF3, interferon regulatory factor 3; JNK, c-Jun N-terminal kinase; MAPK, mitogen-activated protein kinase; MD-2, myeloid differentiation factor 2; MMP, matrix metalloproteinase; MyD88, myeloid differentiation primary response 88; NF-κB, nuclear factor kappa-light-chain-enhancer of activated B cells; RAGE, receptor for advanced glycation end products; sRAGE, soluble RAGE; TIRAP, TIR domain-containing adaptor protein; TLR4, Toll-like receptor 4; TRIF, TIR domain-containing adapter-inducing interferon-β; and VEGF, vascular endothelial growth factor.

Across models, AGE–RAGE-related ligands converge on shared biological programs more than on a single confirmed “RAGE-only” cascade. NF-κB is the most consistently implicated signaling node across HMGB1- and S100A9-centered studies [130,131,136,137] (Figure 8).

Angiogenesis-associated behaviors, including endothelial migration and invasion and vascular marker expression, are repeatedly inducible by RAGE ligand contexts (e.g., HMGB1 stimulation, S100A9-conditioned media) and are attenuated when ligands or upstream triggers are inhibited [129,136].

Fibrotic remodeling emerges as a distinct phenotype linked to RAGE expression. In human ovarian endometrioma tissue, fibrosis correlated strongly with RAGE staining, and analogous positive fibrosis–RAGE correlations were observed in murine lesions as they matured [117]. These results support a temporal framing in which RAGE expression becomes more prominent in chronically remodeled and fibrotic lesions, while inflammatory ligand signals may vary by compartment and stage.

#### 3.2.11. Integration Across Models and Disease Domains 

**Figure 9:**
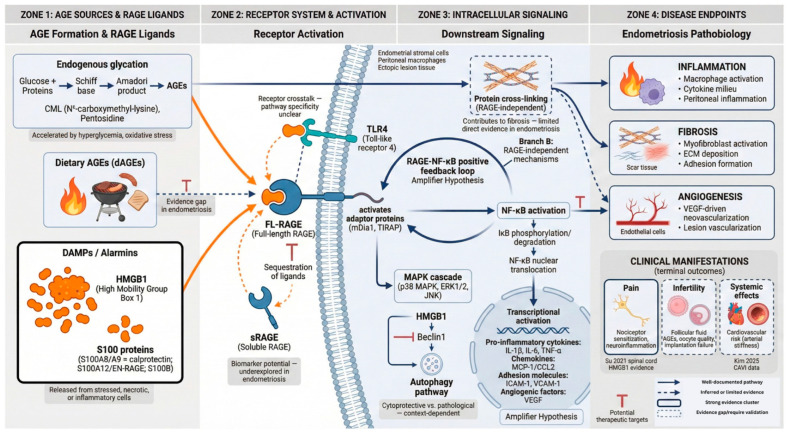
Mechanistic pathway diagram of the AGE–RAGE pathway in endometriosis [122,131]. AGEs arise from endogenous glycation and dietary sources, while damage-associated molecular patterns (HMGB1, S100 proteins) serve as additional RAGE ligands. Receptor activation triggers MAPK and NF-κB signaling cascades, driving pro-inflammatory gene expression. A positive feedback loop (RAGE-NF-κB-RAGE) amplifies inflammatory responses, supporting the role of AGE–RAGE as a disease amplifier rather than initiator. RAGE-independent AGE effects include direct protein cross-linking, contributing to fibrosis. Downstream pathological endpoints include inflammation, fibrosis, angiogenesis, pain, infertility, and systemic cardiovascular effects. Solid lines indicate well-documented pathways; dashed lines indicate inferred or under-investigated connections. Red symbols denote potential therapeutic intervention points. Abbreviations: AGE, advanced glycation end product; CML, Nε-carboxymethyl-lysine; DAMP, damage-associated molecular pattern; ECM, extracellular matrix; HMGB1, high-mobility group box 1; NF-κB, nuclear factor kappa B; RAGE, receptor for advanced glycation end products; sRAGE, soluble RAGE; TLR4, Toll-like receptor 4; and VEGF, vascular endothelial growth factor.

Across human cohorts, patient-derived tissues and cells, in vitro perturbation systems, and animal transplantation models, the AGE–RAGE pathway in endometriosis is most coherently conceptualized as a multifunctional stress–inflammation network rather than a single initiating trigger. AGE burden and RAGE expression provide a permissive scaffold, while ligand availability (especially HMGB1 and S100 family members) and immune-context programming shape outputs, including immune activation or suppression, angiogenesis, fibrosis, metabolic adaptation, reproductive outcomes, and pain sensitization (Figure 9).

## 4. Discussion

In this narrative review, we examined original studies of AGE–RAGE biology in endometriosis. Across 29 studies spanning human cohorts, patient-derived tissues, cell-based systems, and animal models, the evidence suggests that AGE–RAGE signaling more often modifies disease after lesions have formed than it initiates lesion establishment. Increased AGE burden is detectable in selected cohorts [18,119,133]. RAGE and its canonical ligands appear repeatedly in endometriosis-relevant tissues and immune contexts [119,127,130]. However, direct proof that AGE accumulation in lesions is a “first hit” remains sparse. Instead, the findings point toward an amplifier or modifier system. This system shapes inflammation, lesion remodeling, fibrosis, immune phenotypes, infertility-related microenvironments, and pain biology after disease is established [117,124,130,131,136].

This framing has two practical implications. First, it shifts attention from a binary “present versus absent” model toward a context- and stage-dependent model. Second, it aligns with a growing view of endometriosis as a syndrome with distinct expressions. Some forms are mainly inflammatory and associated with pain. Others are more fibrotic and infiltrative. Still, others are enriched for infertility-associated dysregulation [1]. AGE–RAGE components may help explain why similar-looking lesions differ in clinical expression and recurrence. They may also explain why a single treatment approach has variable results across patients.

The current evidence provides only a limited direct measurement of specific AGE adducts in endometriosis tissues. However, existing signals are informative. Fujii et al. (2008) [119] found that CML levels were similar in plasma and follicular fluid. They showed a borderline increase in peritoneal fluid [119]. This supports the idea that AGE enrichment may be localized to the pelvic inflammatory milieu. It does not reflect uniform systemic elevation.

More recent evidence extends this discussion using tissue-based proxies. Smyk et al. reported higher skin AGE autofluorescence in women with endometriosis [18]. Uehara et al. linked serum pentosidine to worsening arterial stiffness over time [133]. These observations do not establish lesion causality. However, they fit the broader clinical picture: endometriosis is linked to cardiometabolic vulnerability and chronic inflammatory stress [18]. The key challenge is determining the role of systemic AGE burden. It may result from chronic disease. It may modify disease course as a parallel exposure. Or it may contribute to lesion biology through local tissue effects.

The strongest tissue-level “structural” signal links RAGE expression to fibrosis. In human ovarian endometrioma samples, lesional fibrosis correlated strongly with RAGE staining [117]. Parallel murine observations suggest that a similar relationship emerges as implants mature. This finding is plausible even if RAGE signaling is not the sole driver. AGE biology can influence tissue remodeling through the direct cross-linking and stiffening of the extracellular matrix. Receptor-related biology can then amplify fibrotic programs through inflammatory and profibrotic mediators.

Importantly, this fibrosis-linked pattern argues against a simplistic view of “more RAGE equals more disease activity”. Rather, RAGE may become most prominent in chronically remodeled lesions. This positions it as a marker, and potentially a mediator, of lesion maturation and structural remodeling.

A second feature supports a maturation model: immune-cell adaptation across disease severity. Shiraishi et al. reported that peritoneal macrophage surface RAGE declines with increasing disease burden [130]. This parallels reductions in other pattern-recognition receptors. In the same study, ex vivo HMGB1 exposure reduced macrophage RAGE/AGER expression. Sustained alarmin exposure may cause receptor desensitization or immune phenotype shifting in advanced disease.

This observation explains a clinical paradox. Endometriosis features persistent inflammation, yet it can also show ineffective lesion clearance and immune tolerance. A model where immune cells undergo progressive receptor remodeling under chronic stimulation could reconcile these features. It could also explain why immunomodulatory interventions may work better at certain disease stages.

A central question, one under-addressed in the literature, is whether phenotypes attributed to “RAGE signaling” are truly RAGE-dependent. Many studies highlight HMGB1 and S100 family proteins as canonical RAGE ligands. However, mechanistic experiments in endometriosis more often implicate TLR4/NF-κB signaling. They rarely test RAGE dependence through receptor blockade or knockdown. This distinction matters. HMGB1 and several S100 ligands can engage multiple pattern-recognition receptors. NF-κB represents a shared convergence node downstream. The practical result is that ligand-driven phenotypes can be robust, even when receptor attribution is uncertain.

A central limitation and critical gap in the current literature is receptor attribution: most evidence is associated with RAGE ligands (e.g., HMGB1, S100 family proteins) and shared downstream nodes (e.g., NF-κB), not a demonstration of RAGE-mediated causality. As a result, many “RAGE signaling” claims remain ligand-driven inferences rather than receptor-resolved mechanisms. Addressing this requires receptor-resolution experiments, such as head-to-head RAGE vs. TLR4 knockdown/knockout or blockade and the use of RAGE-specific antagonists (with appropriate controls) to assign causality to a specific receptor rather than to the ligand or pathway.

The pain literature illustrates this clearly. In a rat model of endometriosis-associated hyperalgesia, pathway analysis emphasized TLR4–MyD88 signaling. Intrathecal blockade reduced neuroinflammatory signaling and pain behaviors [131]. Similarly, Ye et al. studied S100A9-driven macrophage programs and pharmacologic S100A9 inhibition [136]. That study did not determine whether RAGE or TLR4 was the dominant receptor. The conclusion is not that RAGE is irrelevant. Rather, endometriosis models have not yet performed the experiments needed to assign causality to specific receptors.

This receptor ambiguity fits into a coherent, clinically relevant model. We can separate two dimensions of AGE–RAGE biology. One dimension is “structure”: AGE accumulation and AGE-related chemistry can alter matrix properties and tissue stiffness. This can contribute to fibrotic remodeling. The second dimension is “signaling”: Alarmins such as HMGB1 and S100 family members are released in inflammatory, hypoxic, or oxidative environments. They amplify immune and angiogenesis through pattern-recognition receptors. NF-κB is a recurring effector node.

In endometriosis, the signaling dimension is well supported. Mechanistic studies have manipulated ligands or downstream nodes [118,120,131,136]. The structural dimension is suggested most strongly by the fibrosis–RAGE association [117]. It remains less directly tested through measurements of lesion AGE adduct burden and matrix biomechanics. This split may explain a paradox in the literature. There is strong ligand biology but inconsistent evidence for systemic AGE biomarkers. The most relevant pathogenic activity may occur locally. It may manifest as lesion remodeling rather than detectable circulating changes.

A stage-dependent transition from inflammatory amplification to fibrotic remodeling provides a useful lens for integrating disparate findings. Early or active lesions may be driven by inflammatory ligand release, angiogenic activation, and immune-cell recruitment. Later lesions may exhibit increased matrix remodeling, fibrosis, and immune adaptation. Within this model, the directionality of RAGE-related findings becomes easier to interpret. RAGE expression may rise in fibrotic, chronically remodeled lesions [117]. Meanwhile, immune-cell surface PRRs may decline with increasing severity [130].

If correct, this paradigm has treatment implications. Interventions targeting inflammatory ligand signaling may work better in early or highly inflammatory disease. Interventions aimed at fibrosis and tissue remodeling may be more relevant in advanced, stiff, and infiltrative lesions. AGE-related matrix effects would fall into this latter category. Current evidence does not yet allow for definitive staging rules. However, it justifies stage- and phenotype-aware study designs.

The heterogeneity of endometriosis offers a framework to interpret why signals differ across compartments and outcomes. Infertility-associated cohorts illustrate this through the soluble RAGE story. Lin et al. reported that follicular fluid sRAGE was reduced in an IVF cohort enriched for deeply infiltrating endometriosis [124]. sRAGE correlated positively with ovarian response, including oocyte yield and maturity. This pattern is biologically plausible. If sRAGE functions as a local decoy receptor, lower sRAGE could reduce the ligand buffering capacity. This would permit amplified inflammatory signaling in the follicular microenvironment.

In parallel, prospective evidence indicates that innate immune activation involving RAGE ligands may precede clinical diagnosis. Sasamoto et al. reported that elevated pre-diagnostic S100A9 was linked to subsequent endometriosis diagnosis [125]. Associations were stronger closer to diagnosis. These findings do not prove RAGE-specific signaling. However, they strengthen the plausibility of early ligand biology. They underscore the need to link ligand signals to specific clinical phenotypes: recurrence, pain, infertility, and fibrosis.

Beyond the pelvis, systemic signals in some studies connect endometriosis to broader chronic disease biology. Increased skin AGE autofluorescence and the link between pentosidine and worsening arterial stiffness align with the cardiometabolic literature [18,133]. AGE-related burden tracks vascular dysfunction in the literature.

This is an important translational bridge. It addresses a widely observed but incompletely explained phenomenon. Endometriosis is not only a pelvic disease. Its systemic consequences extend into domains typically managed outside gynecology. The implication is not that AGE–RAGE biology explains all systemic risk. Rather, it represents a plausible shared molecular mediator. It links chronic inflammatory metabolic stress to both pelvic lesion behavior and vascular phenotypes.

**Biological plausibility**. The most clinically actionable feature of AGE biology is its modifiability. Unlike fixed genetic risk factors, AGE exposure is partly exogenous. It can be influenced by diet and cooking practices. This is relevant to endometriosis, because patients often seek dietary strategies without definitive clinical guidance. This creates a vacuum often filled by unvalidated interventions.

**Current evidence gap**. No endometriosis-specific dietary AGE intervention trials have been performed. Nonetheless, the broader literature suggests that dietary AGE intake can be reduced by modifying cooking methods. Lower-temperature, higher-moisture techniques are effective. Low-AGE diets can improve metabolic and oxidative stress biomarkers in conditions such as PCOS [138]. The appropriate position is therefore cautious and research forward. Dietary AGE reduction is biologically plausible, low risk, and aligned with patient preferences. However, its effects on endometriosis outcomes are untested. They should be evaluated in rigorous trials.

**Informed speculation**. By analogy to related cardiometabolic conditions in which lowering dietary AGE exposure improves intermediate biomarkers (e.g., oxidative stress and inflammatory readouts), dietary AGE reduction in endometriosis is plausibly more likely to shift systemic inflammation/oxidative stress than to directly reverse established lesions. Accordingly, early trials should prioritize biologically grounded intermediate endpoints (circulating AGE adducts, oxidative stress markers, inflammatory cytokines, and relevant reproductive microenvironment measures where feasible), with symptom outcomes as clinically meaningful co-endpoints. In contrast, complete lesion regression (especially in fibrotic or deeply infiltrating disease) should be treated as an unlikely short-term endpoint and, if assessed, positioned as a longer-term/exploratory outcome rather than an expectation.

A realistic translational agenda should follow the evidence rather than overextend it, and as the top mechanistic priority, receptor-resolution studies are needed to determine whether key phenotypes attributed to “RAGE signaling” are truly RAGE-dependent versus mediated primarily through TLR4 or other PRRs. This includes comparative RAGE vs. TLR4 knockdown/knockout or pharmacologic blockade, and the use of RAGE-selective antagonists, ideally paired with rescue/epistasis designs. Second, lesion phenotype-specific profiling is needed. Ovarian endometrioma is overrepresented in tissue studies. Peritoneal disease and deeply infiltrating endometriosis are less represented despite their clinical importance. Third, longitudinal studies should measure AGE-related markers, RAGE/sRAGE, and ligand profiles, alongside lesion progression and symptom trajectories.

Fourth, receptor dependence must be tested directly. Experiments that selectively block or knockdown RAGE, TLR4, or shared downstream nodes would advance the field. They would move us beyond associative “RAGE ligand” framing toward receptor-resolved mechanistic inference.

Fifth, interventional studies should leverage modifiability. Dietary AGE reduction trials and carefully selected pharmacologic strategies could target AGE formation or neutralize key ligands. These should be linked to biologically grounded endpoints: lesion vascularity, fibrosis, macrophage phenotype, and pain sensitization. This approach would reduce reliance on symptom reports alone.

Limitations and critical gaps constrain interpretation and likely contribute to inconsistency across studies. Most importantly, the evidence base remains largely associative with RAGE ligands rather than causal for RAGE-mediated mechanisms, because receptor-specific blockade/knockdown comparisons (e.g., RAGE vs. TLR4) are rarely performed. Sample sizes are often modest. Menstrual cycle phases and hormonal exposures are inconsistently controlled. Metabolic confounders such as BMI and insulin resistance are variably reported. They are rarely modeled as primary covariates. Control populations range from healthy volunteers to surgical or infertility controls.

In addition, many studies focus on one compartment without paired sampling. These compartments include lesion tissue, peritoneal fluid, follicular fluid, or blood. This limits inference about systemic–local relationships. Finally, the lack of RAGE-specific blockade studies remains a major gap. This is particularly notable, given the frequent framing of results as “RAGE signaling”.

## 5. Conclusions

Across 29 studies, the available evidence positions AGE–RAGE biology as a disease modifier in endometriosis rather than an initiating mechanism. Alarmin-driven signaling shapes inflammation, fibrosis, immune phenotype, pain, and infertility-related microenvironments after lesions are established, but direct demonstrations of AGE accumulation as a causative “first hit” remain sparse. Importantly, at the receptor level, the current evidence base is still largely associative, built around the presence and activity of canonical RAGE ligands and shared downstream pathways (e.g., NF-κB) rather than definitive demonstrations of RAGE-mediated causality.

This framing carries clinical relevance. Endometriosis is increasingly recognized as a heterogeneous syndrome, with some forms being predominantly inflammatory and pain-associated, others fibrotic and infiltrative, and others enriched for infertility-related dysregulation. AGE–RAGE biology may contribute differently across these phenotypes and stages, helping to explain why anatomically similar lesions differ in clinical expression and why uniform treatments yield inconsistent results.

A key mechanistic insight is the distinction between structural effects (matrix cross-linking, tissue stiffening, and fibrotic remodeling) and signaling effects (alarmin-mediated amplification through pattern-recognition receptors). The signaling dimension is well supported experimentally; the structural dimension is suggested by the fibrosis–RAGE association but less directly tested. However, because many “RAGE ligands” are promiscuous DAMPs that also engage other PRRs (notably TLR4), receptor attribution remains uncertain unless receptor dependence is directly tested.

From a translational perspective, AGE exposure is modifiable through diet: An actionable opportunity aligned with patient preferences, though no endometriosis-specific intervention trials exist.

Future progress requires, first and foremost, receptor-resolution experiments that distinguish RAGE-dependent from TLR4-dependent (and shared) mechanisms, using comparative RAGE vs. TLR4 knockdown/blockade and RAGE-specific antagonists in relevant endometriosis models and readouts. Additional priorities include phenotype-specific lesion profiling beyond ovarian endometrioma, longitudinal studies linking AGE-related markers to disease trajectories, and interventional studies tied to biologically grounded endpoints rather than symptom reports alone. By addressing these gaps, beginning with receptor-resolved causality, the field can advance toward the mechanism-informed, phenotype-aware management of endometriosis.

## Figures and Tables

**Table 1:** Characteristics of studies included in the review.

Author	Year	Journal	Study Design	N (E/C)	Phenotype	Biological Samples	Axis Components	Focus
Antsiferova [114]	2013	Biomed Res Int	In Vitro	40/NR	Not specified	PF	RAGE	Mechanistic
Bakun [115]	2024	Wiad Lek	Human	88/68	Infertility-associated	Blood	HMGB1	Infertility
Bakun [116]	2024	Proc Shevchenko Sci Soc	Human	20/10	Infertility-associated	Blood, PF, Tissue	HMGB1, Downstream	Infertility
Cao [117]	2019	Sci Rep	Human, In Vivo	30/20	Ovarian (OMA)	Plasma, Tissue, Animal	RAGE, HMGB1, Downstream	Mechanistic
Chen [118]	2023	J Biomed Res	Human, In Vitro	10/10	Not specified	Tissue	HMGB1, Downstream, Intervention	Mechanistic
Fujii [119]	2008	Reprod Sci	Human	NR/NR	Ovarian (OMA)	Plasma, PF, FF, Tissue	AGE, RAGE, Downstream	Infertility
Huang [16]	2021	Front Endocrinol	Human, In Vitro	58/20	Ovarian (OMA)	Tissue, Primary Cells	HMGB1, Downstream, Intervention	Infertility
Huang [11]	2022	Int J Mol Sci	Human, In Vitro, In Vivo	28/15	Ovarian (OMA)	Serum, Tissue, Primary Cells, Cell Line	HMGB1	Mechanistic
Huang [120]	2024	Int Immunopharmacol	Human, In Vitro, In Vivo	28/9	Ovarian (OMA)	Tissue, Primary Cells, Animal	HMGB1, Downstream, Intervention	Mechanistic
Ikeda [121]	2021	J Reprod Immunol	Human, In Vitro	14/8	Ovarian (OMA)	Serum, Tissue	RAGE, HMGB1, Downstream, Intervention	Mechanistic
Kim [122]	2025	J Clin Med	In Vitro	NR/NR	Infertility-associated	Other	HMGB1, Downstream	Infertility
Li [123]	2022	Bioengineered	In Vitro	NR/NR	Not specified	Primary Cells	HMGB1, Downstream, Intervention	Mechanistic
Lin [124]	2020	Redox Biol	Human, In Vitro, In Vivo	131/127	DIE	FF, Tissue, Cell Line, Animal	RAGE	Infertility
Perricos [17]	2023	Int J Mol Sci	Human	51/30	Ovarian (OMA)	Plasma, PF, Animal	S100	Mechanistic
Sasamoto [125]	2025	eBioMedicine	Human	200/200	Ovarian (OMA)	Plasma	S100	Mechanistic
Sharma [126]	2010	Fertil Steril	In Vitro	15/10	Ovarian (OMA)	Tissue, Primary Cells	RAGE, S100	Mechanistic
Sharma [127]	2010	Int J Gynaecol Obstet	Human	28/20	Ovarian (OMA)	Tissue, Primary Cells	RAGE, S100, Downstream	Infertility
Sharma [128]	2011	Fertil Steril	In Vitro	15/10	Ovarian (OMA)	Tissue, Primary Cells	RAGE, S100	Mechanistic
Shimizu [129]	2017	Reprod Sci	Human, In Vitro	84/55	Ovarian (OMA)	Serum, PF, Tissue, Primary Cells	RAGE, HMGB1, Downstream	Mechanistic
Shiraishi [130]	2024	J Reprod Immunol	Human	27/6	Ovarian (OMA)	PF	RAGE, HMGB1, S100, Downstream, Intervention	Mechanistic
Smyk [18]	2024	Sci Rep	Human	21/24	Ovarian (OMA)	Other	AGE, Downstream	Cardiovascular
Su [131]	2021	J Neuroinflammation	In Vivo	NR/NR	Mixed/Other	Other	HMGB1, Downstream, Intervention	Pain
Sun [132]	2023	Cell Mol Life Sci	Human, In Vitro, In Vivo	50/50	Ovarian (OMA)	PF, Tissue, Primary Cells, Cell Line	HMGB1, Downstream, Intervention	Mechanistic
Uehara [133]	2022	J Obstet Gynaecol Res	Human	207/NR	Mixed/Other	Serum	AGE	Cardiovascular
Wang [134]	2021	PeerJ	In Silico, In Vitro	NR/NR	Not specified	Tissue, Primary Cells, Cell Line	Downstream	Mechanistic
Wei [135]	2025	J Obstet Gynaecol Res	Human, In Vitro, In Vivo	11/6	Ovarian (OMA)	PF, Tissue, Animal	HMGB1, Downstream, Intervention	Mechanistic
Ye [136]	2025	Mol Hum Reprod	Human, In Vitro, In Vivo	NR/NR	Ovarian (OMA)	Other	S100, Downstream, Intervention	Mechanistic
Yun [137]	2016	PLoS ONE	Human, In Vitro	33/37	Not specified	Tissue, Primary Cells	RAGE, HMGB1, Downstream	Mechanistic
Zhu [138]	2020	Syst Biol Reprod Med	Human	56/37	Ovarian (OMA)	Tissue	S100	Mechanistic

Abbreviations: AGE, advanced glycation end products; CML, Nε-carboxymethyllysine; DIE, deeply infiltrating endometriosis; E/C, endometriosis/control; FF, follicular fluid; HMGB1, high-mobility group box 1; NR, not reported; PF, peritoneal fluid; RAGE, receptor for advanced glycation end products.

## Data Availability

No new data were created or analyzed in this study. Data sharing is not applicable to this article.

## Supplementary Materials

The following supporting information can be downloaded at: https://www.mdpi.com/article/10.3390/ijms27031396/s1.
