# Supplementary material for: The AGE–RAGE Pathway in Endometriosis: A Focused Mechanistic Review and Structured Evidence Map
Source: Int J Mol Sci. 2026 Jan 30;27(3):1396. doi: 10.3390/ijms27031396 (PMC12898717; doi:10.3390/ijms27031396)
Supplement: Supplementary file 1 [file ijms-27-01396-s001.zip › SD1.pdf]

PRISMA 2020 Checklist

The AGE-RAGE pathway in endometriosis: a focused mechanistic review and structured evidence map

Note: This review follows a systematic-narrative hybrid methodology (Turnbull et al., 2023). Items marked 'Not applicable' reflect the narrative synthesis approach.

Section and Topic	Item #	Checklist Item	Location where item is reported
**TITLE**<br>Title	1	Identify the report as a systematic review.	Title page: 'focused mechanistic review and structured evidence map'
**ABSTRACT**<br>Abstract	2	See the PRISMA 2020 for Abstracts checklist.	Abstract, page 1
**INTRODUCTION**<br>Rationale	3	Describe the rationale for the review in the context of existing knowledge.	Introduction, paragraphs 1–5
Objectives	4	Provide an explicit statement of the objective(s) or question(s) the review addresses.	Introduction, final paragraph
**METHODS**<br>Eligibility criteria	5	Specify the inclusion and exclusion criteria for the review and how studies were grouped for the syntheses.	Materials and Methods, 'Eligibility Criteria'
Information sources	6	Specify all databases, registers, websites, organizations, reference lists and other sources searched or consulted to identify studies.	Materials and Methods, 'Search Strategy'
Search strategy	7	Present the full search strategies for all databases, registers and websites, including any filters and limits used.	Materials and Methods, 'Search Strategy' (full strings provided)
Selection process	8	Specify the methods used to decide whether a study met the inclusion criteria of the review.	Materials and Methods, 'Screening and Selection'
Data collection process	9	Specify the methods used to collect data from reports.	Materials and Methods, 'Data Extraction and Synthesis'
Data items	10a	List and define all outcomes for which data were sought.	Materials and Methods, 'Data Extraction and Synthesis'
	10b	List and define all other variables for which data were sought.	Materials and Methods, 'Data Extraction and Synthesis'
Study risk of bias assessment	11	Specify the methods used to assess risk of bias in the included studies.	Materials and Methods, 'Risk of Bias and Quality Assessment' - formal assessment not performed; rationale provided
Effect measures	12	Specify for each outcome the effect measure(s) used in the synthesis or presentation of results.	Not applicable - narrative synthesis without meta-analysis
Synthesis methods	13a	Describe the processes used to decide which studies were eligible for each synthesis.	Materials and Methods, 'Data Extraction and Synthesis'
	13b	Describe any methods required to prepare the data for presentation or synthesis.	Not applicable
	13c	Describe any methods used to tabulate or visually display results of individual studies and syntheses.	Materials and Methods; Results, Figure 3 and Table 1

Section and Topic	Item #	Checklist Item	Location where item is reported
	13d	Describe any methods used to synthesize results and provide a rationale for the choice(s).	Materials and Methods - narrative synthesis; rationale provided
	13e	Describe any methods used to explore possible causes of heterogeneity among study results.	Not applicable - narrative synthesis
	13f	Describe any sensitivity analyses conducted to assess robustness of the synthesized results.	Not applicable - narrative synthesis
Reporting bias assessment	14	Describe any methods used to assess risk of bias due to missing results in a synthesis.	Not formally assessed; acknowledged in Discussion
Certainty assessment	15	Describe any methods used to assess certainty (or confidence) in the body of evidence for an outcome.	Materials and Methods, 'Certainty of Evidence' - GRADE not applied; narrative assessment described
**RESULTS** Study selection	16a	Describe the results of the search and selection process, from the number of records identified in the search to the number of studies included in the review, ideally using a flow diagram.	Materials and Methods, 'Screening and Selection'; Supplementary Document 1 (flow diagram)
	16b	Cite studies that might appear to meet the inclusion criteria, but which were excluded, and explain why they were excluded.	Exclusion categories provided; individual citations not listed
Study characteristics	17	Cite each included study and present its characteristics.	Results; Table 1; Supplementary Document 3
Risk of bias in studies	18	Present assessments of risk of bias for each included study.	Not performed - rationale in Materials and Methods
Results of individual studies	19	For all outcomes, present, for each study: (a) summary statistics for each group and (b) an effect estimate and its precision.	Results sections; Supplementary Document 3 (data extraction)
Results of syntheses	20a	For each synthesis, briefly summarise the characteristics and risk of bias among contributing studies.	Results - characteristics integrated into narrative
	20b	Present results of all statistical syntheses conducted.	Not applicable - no meta-analysis performed
	20c	Present results of all investigations of possible causes of heterogeneity among study results.	Results - heterogeneity discussed narratively by model system
	20d	Present results of all sensitivity analyses conducted to assess the robustness of the synthesized results.	Not applicable
Reporting biases	21	Present assessments of risk of bias due to missing results for each synthesis assessed.	Discussion, limitations section
Certainty of evidence	22	Present assessments of certainty in the body of evidence for each outcome assessed.	Discussion - evidence certainty discussed narratively
**DISCUSSION** Discussion	23a	Provide a general interpretation of the results in the context of other evidence.	Discussion, throughout

Section and Topic	Item #	Checklist Item	Location where item is reported
	23b	Discuss any limitations of the evidence included in the review.	Discussion, limitations paragraphs
	23c	Discuss any limitations of the review processes used.	Discussion, limitations paragraphs
	23d	Discuss implications of the results for practice, policy, and future research.	Discussion; Conclusion
**OTHER INFORMATION** Registration and protocol	24a	Provide registration information for the review, including register name and registration number, or state that the review was not registered.	Materials and Methods, 'Protocol Registration' - not registered. Narrative review.
	24b	Indicate where the review protocol can be accessed, or state that a protocol was not prepared.	Materials and Methods - formal protocol not prepared
	24c	Describe and explain any amendments to information provided at registration or in the protocol.	Not applicable
Support	25	Describe sources of financial or non-financial support for the review, and the role of the funders or sponsors in the review.	Acknowledgments/Funding section
Competing interests	26	Declare any competing interests of review authors.	Conflict of Interest statement
Availability of data, code and other materials	27	Report which of the following are publicly available and where they can be found: template data collection forms; data extracted from included studies; analytic code; any other materials used in the review.	Data Availability statement; Supplementary Document 3

Reference: Page MJ, McKenzie JE, Bossuyt PM, et al. The PRISMA 2020 statement: an updated guideline for reporting systematic reviews. BMJ 2021;372:n71.

Methodology: Turnbull D, Chugh R, Luck J. Systematic-narrative hybrid literature review. Social Sciences & Humanities Open. 2023;7(1):100381.
